# Has Zika been forgotten? A qualitative exploration of knowledge gaps, perceived risk and preventive practices in pregnant women in Malaysia

**DOI:** 10.1186/s12905-024-02999-z

**Published:** 2024-03-21

**Authors:** Li Ping Wong, Haridah Alias, Hai Yen Lee, Sazaly AbuBakar, Yulan Lin, Zhijian Hu

**Affiliations:** 1 Fujian Key Laboratory of Environmental Factors and Cancer, Department of Epidemiology and Health Statistics, School of Public Health, Fujian Medical University, Fuzhou, 350122 Fujian Province China; 2 Centre for Epidemiology and Evidence-Based Practice, Department of Social and Preventive Medicine, Faculty of Medicine, Universiti Malaya, 50603 Kuala Lumpur, Malaysia; 3 Tropical Infectious Diseases Research and Educational Centre (TIDREC), Universiti Malaya, 50603 Kuala Lumpur, Malaysia

**Keywords:** Asian women, Infectious disease, Mosquito, Safe pregnancy, ZIKV

## Abstract

**Background:**

Although Zika virus infection is rarely reported now, continuous prevention is needed to achieve sustained eradication. This study aimed to explore the knowledge gaps, risk perception and preventive measures against Zika virus infection (ZIKV) in pregnant women in Malaysia.

**Methods:**

We conducted in-depth virtual interviews with pregnant women between February and April 2022. The interviews were recorded and transcribed, and data were analyzed by *c*ontent analysis.

**Results:**

The majority of the participants demonstrated a commendable level of awareness regarding the signs and symptoms associated with ZIKV infection. They also exhibited a clear understanding of preventive measures, particularly emphasizing the importance of avoiding mosquito bites to minimize the risk of ZIKV transmission. However, a noteworthy gap in knowledge surfaced as a subset of participants remained uninformed about the potential for sexual transmission of ZIKV, which could lead to congenital ZIKV in pregnant women. Even among women who were cognizant of ZIKV and its potential negative health outcomes, associated with the infection, many of them did not perceive themselves to be at risk, mainly because ZIKV infection is infrequently discussed or heard of, leading to a sense of infections’ rarity. While the adoption of preventive measures such as mosquito bite prevention during pregnancy was a common practice, however, prevention of sexually transmitted infections (STIs) including mosquito-borne diseases such as Zika is low. A minority of women express concerns about the sensitivity surrounding discussions and prevention of STIs within the context of marriage. Most of the participants were supportive of the provision of awareness of ZIKV infection in women during pregnancy and the involvement of men, especially in initiatives aimed at preventing transmission through sexual contact.

**Conclusion:**

This study uncovered gaps in both knowledge and practices pertaining ZIKV infection among pregnant women in the aftermath of the ZIKV pandemic. The insights gleaned from our research are valuable for shaping future interventions geared towards preventing the resurgence or facilitating the sustainable eradication of ZIKV.

**Supplementary Information:**

The online version contains supplementary material available at 10.1186/s12905-024-02999-z.

## Background

Zika virus (ZIKV) infection is an acute exanthematous disease transmitted to humans primarily through the bite of an infected Aedes species mosquito [1]. ZIKV infection typically causes dengue-like symptoms including mild fever, rash, arthritis, headache, conjunctivitis and oedema [2]. Infection during pregnancy can cause microcephaly and other congenital abnormalities in the developing foetus and newborn, and may also result in pregnancy complications such as foetal loss, stillbirth, and premature birth [3]. ZIKV was considered a benign disease until October 2015, when a sharp increase in the number of neonates born with microcephaly, was observed in Brazil [4]. On 1 February 2016, the World Health Organization (WHO) declared Zika a public health emergency of international concern [5].

The Zika pandemic raises concerns in Malaysia, given the country's hyperendemicity with dengue and the presence of favorable ecological conditions for ZIKV transmission, including warm temperatures, humidity, and high rainfall [6, 7]. Although Malaysia has never had a ZIKV outbreak, the history of ZIKV infection in Malaysia dates to 1969 when the first Zika virus was isolated in *Aedes aegypti* [8]. ZIKV transmission in Malaysia has been reported before the WHO declared Zika a public health emergency of international concern in 2016. In 2014, ZIKV was diagnosed in a traveler from Malaysia [9]. During the pandemic of ZIKV in 2016, a total of eight cases of Zika were confirmed in Malaysia [10]. Nevertheless, no confirmed cases of Zika were reported in 2017 and 2018. While the epidemic had subsided in Malaysia, a case of Zika virus (ZIKV) was detected in October 2019 [11].

Even though Zika no longer being a Public Health Emergency of International Concern since 2016, Malaysia maintains a comprehensive strategy to prevent its transmission. National, state, and district-level surveillance continues, constantly on the lookout for potential instances of microcephaly and Guillain-Barré syndrome [12]. In Malaysia, reporting suspected and confirmed Zika cases, microcephaly, and Guillain-Barré syndrome is mandatory. Positive Zika cases prompt immediate vector control within a 400-m radius of the index case's residence, with active case detection among household members, close contacts, and neighbors in affected areas. Malaysia's strategy for Zika management emphasizes surveillance, prompt reporting, vector control, and community engagement. Travelers from countries with Zika virus infections meeting case definitions are referred to health facilities for appropriate measures.

Despite the overall decrease in cases, Malaysia remains cautious about the reemergence of the ZIKV, as new instances persist in neighboring countries such as Vietnam [13] and, more recently, in the Lao People’s Democratic Republic in 2020 [14]. Globalization, the explosion of international trade and travel is well known as one factor in the spread of ZIKV [15]. Malaysia should be concerned about the continued presence of ZIKV in neighboring countries due to regional connectivity, trade, and travel, which make it easier for the virus to spread across borders and enter the country, putting Malaysian population at risk.

Currently, ZIKV vaccine is not yet available for public use. However, several ZIKV vaccine candidates are in different stages of preclinical and clinical development [16]. Therefore, the best means of prevention is to avoid the transmission of ZIKV through measures such as mosquito bite prevention, safe sexual practices, and adhere to travel advisories in regions where the virus is prevalent. Although *Aedes* spp. mosquitoes are the principal vector responsible for the widespread transmission of ZIKV, it can be transmitted between humans through sexual contact [17]. The sexual transmission of ZIKV carries significant implications for pregnant women, especially those residing in endemic regions, as well as for women whose spouses or sexual partners are traveling to or returning from areas with active ZIKV transmission.

Despite the rare occurrence of reported ZIKV infections, persistent prevention efforts are crucial for achieving sustained eradication. It is imperative to assess whether pregnant women are aware of the ZIKV risk and actively practicing preventive measures. Given the significance of safe sex practices in preventing sexual transmission of ZIKV, it is essential to thoroughly investigate barriers to preventing Zika during pregnancy.

Although pregnant women are at exceptional risk of being infected with ZIKV infection which is a known cause of microcephaly and other congenital and developmental anomalies, no research has explored in-depth their knowledge gaps, prevention practices and barriers to prevention, particularly in preventing sexual transmission of ZIKV in Malaysia. In light of the limited in-depth exploration, this study aimed to explore the knowledge gaps, risk perception and preventive measures against Zika virus infection in pregnant women in Malaysia. By doing so, our findings can contribute to the development of effective public health strategies to prevent Zika virus infection among pregnant women in Malaysia and beyond.

## Methods

### Sampling

A purposive sample of women who met the eligibility criteria, which is being currently pregnant, and having no pre-existing comorbidities, were recruited for the study. Recruitment of participants was done through several methods. The study was conducted in collaboration with a community organization and a women's health clinic in the Klang Valley, Selangor, an urban conglomeration centered in the federal territory of Kuala Lumpur, Malaysia. This collaboration was crucial for facilitating the recruitment of pregnant women, given the sensitivity of discussions surrounding this issue. The decision to focus on the Klang Valley was purposeful, driven by the goal of encompassing region experiencing significant population and economic growth. Further, Selangor has the highest concentration of dengue cases [18], underscoring the importance of studying dengue related issues in this specific geographic context, where dengue continues to persist.

We also use online platforms and social media to advertise our interview to reach a wider audience. The recruitment process also utilized online platforms and social media, using snowballing techniques. The study information was disseminated through these channels, reaching a diverse audience. If individuals expressed interest after seeing the advertisement, they were instructed to reach out the research team using the provided contact information. Once contacted, we assessed whether they met the inclusion criteria, explained the study's objectives and method details, and arranged for an interview. Prior to participation, participants were provided with detailed information, and informed consent was obtained. All participants were compensated with a monetary token for their time dedicated to participating in the study.

### Data collection

The data were collected through qualitative research interviews conducted by a single researcher who is a member of the research team. Using a sole interviewer ensures consistency in the data collection process, including adherence to the interview guide, consistent probing, and a smoother flow of discussion. As a member of the research team, the interviewer has an in-depth understanding of the study objectives, research questions, and the context in which the interview takes place. A semi-structured interview guide (Appendix 1) was employed to explore participants' knowledge and risk perception of ZIKV, their prevention practices against ZIKV during pregnancy, and their perceived needs and preferred modes of health promotion regarding the prevention of sexual transmission of ZIKV. The research team members developed the interview guide, which underwent validation by panel experts. Subsequently, it was pilot-tested before the actual commencement of the study.

Conducted amidst the COVID-19 pandemic, the study opted for virtual interviews facilitated through video chat for data collection. The process involved mutual agreement between the interviewer and participants regarding a suitable interview time. Participants were provided with an online Zoom link and a specific schedule for the interview. The interview sessions typically lasted between 30 to 45 min. The arrangement for data collection prioritized the convenience of participants, and interviews were conducted either during office hours or in the evening, ensuring flexibility and accessibility for all involved. Following the interviews, demographic information of the participants was gathered. Interviews were audio-recorded and transcribed verbatim. Interviews were conducted in English and Bahasa Malaysia (the national language in Malaysia). Interviews conducted in Bahasa Malaysia are translated into English by a qualified translator who is fluent in both languages. The translated English versions of the interviews underwent review by two research team members proficient in both Bahasa Malaysia and English. They meticulously compared the translated text with the original interviews to identify any potential discrepancies, inaccuracies, or loss of meaning during the translation process. The data collection process continued until data saturation was attained, determined by criteria such as the repetition of themes, the emergence of consistent patterns, and the absence of new interview data offering additional insights. Saturation was observed after the completion of seven interviews, as no novel themes were identified. The data collection period spanned from February to April 2022.

Ensuring the confidentiality of data was of utmost importance in this study. All video and audio recordings were meticulously stored in a password-protected database, accessible only to two designated research team members. Following the successful completion of the study, all recorded video and audio materials will be securely stored in a cloud platform with password protection. As per organizational guidelines, these materials will be retained for a minimum of five years before being considered for disposal. This approach is consistent with our organization's practices and underscores our commitment to safeguarding the confidentiality and integrity of the collected data.

### Data analysis

The data were analyzed using the conventional content analysis method and NVivo software (version 10.0). An initial set of codes and themes were defined to correspond to the research objectives. Additional codes and themes were subsequently developed through review of transcripts with research team members. Simultaneous data collection and analysis were undertaken, led by two researchers from the research team. The inclusion of two researchers in the analysis facilitates cross-validation of findings. The two researchers compare their interpretations, discuss various perspectives, and collaboratively make decisions concerning coding, themes, and interpretations derived from the data.

This study implemented rigorous measures to ensure trustworthiness. To maintain rigor and transparency in data analysis, themes were independently identified to mitigate potential bias, and inter-coder reliability was established. When interpreting and analyzing the evidence, we remained conscious of the assumptions underlying our perspective, refraining from making any assumptions about participants' viewpoints.

### Ethical approval

Ethical approval was obtained from the University of Malaya Research Ethics Committee (approval code: UM.TNC2/UMREC-586). Participation in this study is entirely voluntary, and participants were fully briefed on the research goals and methodology. Assurance was given regarding the maintenance of confidentiality, and all responses were treated with utmost privacy. Written informed consent was obtained from each participant. The participation information sheet and informed consent form were dispatched to participants through WhatsApp or email, allowing them to review, sign, and electronically return the documents to the researcher.

## Results

Table 1 provides an overview of the participants’ demographic profile. Out of the seven participants, the average age was 30.3 years, with the majority having completed college. The gestational age of pregnant women included in this research ranged from 9 to 34 weeks.

**Table 1:** Demographic characteristics of the participants (*N* = 7)

	Socio demographic characteristics					Pregnancy information		Experience with mosquito bites during pregnancy		Sexual activity during pregnancy	
Participant	Age	Ethnicity	Religion	Qualification	Occupation	Number of pregnancies	Gestational age (weeks)	Frequency bitten by the mosquitoes in the house	Frequency bitten by the mosquitoes outside the house	Frequency of having sexual intercourse during pregnancy	Frequency of having sexual intercourse without protection during pregnancy
1	37	Bumiputera Sabah	Islam	Tertiary	Officer	Second	9	Sometimes	Sometimes	Often	Often
2	29	Malay	Islam	Tertiary	IT Consultant	Second	16	Seldom	Seldom	Sometimes	Sometimes
3	29	Malay	Islam	Tertiary	Clerk	Second	10	Seldom	Seldom	Often	Often
4	26	Malay	Islam	Tertiary	Flight Attendant	First	34	Sometimes	Sometimes	Sometimes	Sometimes
5	29	Malay	Islam	Tertiary	Content Moderator	First	16	Sometimes	Sometimes	Sometimes	Sometimes
6	29	Chinese	Buddha	Tertiary	Clinical Research Associate	First	20	Seldom	Seldom	Often	Often
7	33	Malay	Islam	Tertiary	Housewife	Second	19	Seldom	Seldom	Sometimes	Often

Only one participant reported being completely unaware or had never heard of ZIKV infection. Among those who have heard of ZIKV, many (six out of seven) are aware that the symptoms of ZIKV infection are similar to dengue fever. Many were able to name the common symptoms of ZIKV, such as fever, headache, muscle and joint pain. In total, three participants had heard of ZIKV but were unaware that ZIKV infection during pregnancy is a cause of congenital abnormalities. Most of the participants had heard about ZIKV from the news media in the year 2016, during which the WHO declared Zika a public health emergency of international concern. One participant reported that she had seen a brochure about ZIKV during her antenatal visits. In subsequent interviews, when probed, none of the remaining participants reported ZIKV flyers being available at the gynaecology clinics that they attended. One participant noted that she came across ZIKV infection while browsing on social media.

“*Social media is a powerful tool to educate people. Most of us are on social media. I accidentally came across Zika virus when I was browsing through social media*.”

Regarding the mode of transmission and prevention of ZIKV, most women were aware that ZIKV infection is a mosquito-borne infection. All were very knowledgeable on self-protection methods against mosquito bites, removal or preventing mosquito breeding sites. Most participants also reported that during pregnancy they are particularly conscientious in carrying out the prevention of mosquito-borne infections, including eliminating mosquito breeding sites and avoiding mosquito bites. However, due to ZIKV infection being almost unheard of today, all participants noted that the mosquito preventions they carried out were due to fear of dengue rather than ZIKV infection.

“*I always protect myself against mosquito bites, especially during pregnancy, but it is because I am afraid of getting dengue*.”

“*Zika is not heard of now, no cases in Malaysia or near us, so I don’t think we are at risk*.”

Among those who were aware of ZIKV's sexual transmission, many believed they faced a low risk of contracting it from their spouses or sexual partners. None of the participants had considered practicing condom use during sexual intercourse or abstaining from sex throughout their pregnancy to prevent sexual transmission of ZIKV. This was due to various reasons, such as their belief that their spouses were not at high risk of mosquito-borne infection or that they lived in areas without active local transmission of ZIKV. However, some participants chose to practice abstinence or use condoms during sexual intercourse because they believed that unprotected sex might harm fetal development.

“*My husband does not work in a place with many mosquitoes or travel to places, so it crossed my mind to practice safe sex. Nonetheless, clearing mosquito breeding places, installing mosquito nets, aerial spraying and using mosquito repellent spray are practices that we usually carry out to prevent mosquito bites*.”

“*Semen is not good for the foetus, and sex during pregnancy may hurt the foetus, especially during the early stage of pregnancy, we do not have sexual intercourse and if we did we practise safe sex, but not to prevent sexually transmitted diseases*.”

All participants reported that they encountered no difficulties in abstaining from sex during their pregnancy or using condoms during sexual intercourse to protect themselves against sexual transmission of ZIKV. The reason for this was that their spouses were very understanding and willing to discuss safe sex practices, including the prevention of sexually transmitted infections (STIs). Only two women felt that it was somewhat sensitive to initiate discussions about abstaining from sex during pregnancy or using condoms during sexual intercourse, and suggested that educating spouses of pregnant women about sexual transmission during pregnancy when they accompany their partners on antenatal visits could be helpful. However, most participants agreed that men who are informed about sexual transmission during pregnancy are more likely to support their partners in practicing safe sex during pregnancy. Many believed that if men had awareness of the issue, it would be simpler to discuss abstinence or safe sex practices during pregnancy.

“*I don’t feel sensitive because it is for the sake of our baby, my husband is very understanding and I don’t anticipate any problem if I tell him that we should practice safe sex to prevent Zika transmission during pregnancy*.”

“*I don’t think men in general, would mind practicing safe sex to prevent transmission to the foetus*.”

All women reported that healthcare providers did not provide information about the risks of STIs and their potential consequences, such as pregnancy complications and congenital deformities. Instead, the primary advice given during antenatal check-ups focused on healthy lifestyle choices and nutrition. When questioned further, participants expressed comfort with receiving awareness about STI prevention from healthcare workers during antenatal visits and did not feel stigmatized or uneasy about it.

“*During antenatal visits, we were educated about healthy eating and balanced nutrition, but we were never told about the risk of sexually transmitted diseases during pregnancy or about Zika*.”

“*I think healthcare providers should educate pregnant women about sexual transmission of Zika and protective behaviours*.”

When asked for their thoughts on how to raise awareness about the risk of sexual transmission during pregnancy, many participants stressed the importance of incorporating education on STIs into early antenatal care visits. They believed that making such education a standard part of prenatal care would help ensure that expectant mothers receive the information they need to make informed decisions about their sexual health. Table 2 shows the summary of themes and applicable quotes based on the interviews.

**Table 2:** Summary of themes and applicable quotes based on the interviews

	Themes	Summary of quotes
Knowledge gaps	Source of information	Social media, flyers in clinics
	Knowledge about Zika	Women surveyed were knowledgeable about self-protection methods against mosquito bites and removing mosquito breeding sites, and during pregnancy, they were diligent in implementing these measures to prevent mosquito-borne infections
Risk perception	Risk Perception	Minimal concern regarding Zika as it is rarely discussed
		Neither the women nor their spouses perceived themselves to be at high risk for ZIKV infection, because they believed that they were not in a location where the virus posed a high risk
Preventive measures	Prevention of mosquito bite	High awareness that ZIKV infection is transmitted through mosquito bites, and were knowledgeable about self-protection methods and eliminating mosquito breeding sites
		Often took precautions against mosquito bites, but not because of concerns about Zika, but rather out of a general fear of contracting dengue fever
	Preventing sexual transmission of Zika	While women practice sexual protection during pregnancy, their primary concern was not specifically protecting against ZIKV infection, but rather practicing general sexual protection measures

## Discussion

Specifically, the study sought to assess the need for health promotion efforts focusing on the sexual transmission of ZIKV and to determine which prevention measures can be effectively implemented in current antenatal care settings. By providing valuable insights into these areas, the study's findings can inform strategies to enhance ZIKV prevention and control efforts during pregnancy.

In this study, the majority of women who were familiar with ZIKV knew that it is a mosquito-borne disease and recognized its similarities to dengue fever, which is endemic in Malaysia [19]. Given the history of dengue outbreaks in the country, dengue awareness campaigns are regularly conducted, contributing to a generally high level of knowledge among the study participants regarding mosquito prevention measures. This existing knowledge and awareness of mosquito-borne diseases may have influenced the participants' responses regarding ZIKV prevention practices.

Pregnant women in this study were aware of ZIKV from various sources, and the findings have implications for how to effectively educate women about ZIKV. Firstly, during the early stages of the ZIKV epidemic in 2016, the virus received extensive media coverage in Malaysia, which led to increased awareness among study participants. This suggests that the news media play a significant role in disseminating information about ZIKV. Secondly, the antenatal clinic serves as a source of pregnancy and birth-related information, including ZIKV. Thus, healthcare providers have an opportunity to educate pregnant women about ZIKV and its associated health risks during antenatal care visits. Moreover, making ZIKV-related information readily available in healthcare facilities can further augment awareness among pregnant women. Thirdly, social media platforms can be leveraged to create awareness of ZIKV in this population. As pregnant women frequently use social media to fulfill various pregnancy-related needs or seek information on parenting, utilizing these platforms to disseminate information about ZIKV can be an effective strategy. Indeed, studies have demonstrated that social media can be a potent tool in raising awareness of infectious diseases and influencing behavior change [20–22].

Likewise, a recent public survey in Malaysia found that the general public perceives a low risk of getting ZIKV and are not concerned about it [23]. This is consistent with the findings of this study, where the perceived low risk is primarily due to the rarity of ZIKV cases. Therefore, it is crucial to educate pregnant women about the potential threat of ZIKV. With respect to prevention practices, while many of our study participants reported practicing abstinence or using condoms during sexual intercourse, the motivation behind this was not to prevent sexually transmitted ZIKV or other STIs, but rather to avoid harming the developing fetus. An earlier study found that preventive measures such as abstinence or condom use in the context of a monogamous relationship were not well received by pregnant women and their partners [24]. However, when our study participants were informed about the potential risk of sexual transmission of ZIKV, they expressed willingness to adopt abstinence or use condoms to protect against ZIKV during pregnancy. It appears that a minority of women in this study are sensitive to the issue of preventing STIs in a marital context. This suggests the need to assess whether pregnant women or couples have concerns about sexual protection against ZIKV during pregnancy and to provide subsequent counseling or assistance in communicating appropriately between couples.

Based on the results of this this and various global studies, it is imperative to provide pregnant women with education regarding the risk of acquiring ZIKV and emphasize the significance of safeguarding themselves from potential sexual transmission. In Brazil, research revealed that pregnant women had limited awareness of how ZIKV can be transmitted through sexual contact with an infected person, and only around 20% of them practiced safe sex during pregnancy [25]. Similarly, a study in the Caribbean found that over 70% of pregnant women were unaware of the connection between sexual transmission and ZIKV [26]. Moreover, a study involving pregnant women in Greece discovered that nearly two-thirds (63.3%) of participants had insufficient knowledge regarding sexual transmission of Zika, while almost a quarter (24.1%) were uninformed about the associated risks for the developing fetus and newborn [27]. These findings underscore the necessity for increased education and awareness programs aimed at pregnant women to ensure they take appropriate measures to safeguard their health and that of their unborn children.

Overall, women tend to value guidance from healthcare providers regarding the sexual transmission and prevention of ZIKV during pregnancy. They do not feel embarrassed receiving information related to sexual health during pregnancy, as it affects critical issues related to pregnancy outcomes for both the mother and child. Our study findings suggest that healthcare providers play a vital role in routine counseling on the prevention of sexually transmitted diseases during antenatal visits. Future further studies are warranted to uncover the lack of advice from healthcare workers during antenatal visits regarding the risk of STIs during pregnancy. STI-related stigma and shame may deter patient–provider discussion about STIs in healthcare facilities [28, 29]. Providing communication training for healthcare providers could help them engage pregnant women in conversations about safe sex practices during pregnancy. Behavioral counseling interventions have been shown to be effective in reducing the incidence of STIs [30], underscoring the importance of incorporating such interventions into routine antenatal care.

An important highlight of this study is that women prefer their spouses to receive education from healthcare providers about the sexual transmission and prevention of ZIKV during pregnancy. Our findings indicate that involving men in the prevention of ZIKV during pregnancy is a crucial strategy for preventing the sexual transmission of ZIKV. Worldwide, men's participation and involvement in maternal health have been linked to favorable reproductive health outcomes [31]. Consistent with our findings, a previous study revealed that concurrent receipt of the HIV/STI prevention intervention by women and their partners yielded greater efficacy compared to instances where women received the intervention independently [32]. Similarly, another study reported that couple-centered approaches may prove more effective in addressing the STI prevention [33].

First and foremost, this study has the limitation of being a qualitative study, which limits its ability to be generalized to larger populations. Another limitation is that, despite reaching data saturation, the study was unable to capture responses from women with dominant spouses who face challenges in negotiating safe sexual practices with their partners. Additionally, all women in the study had a tertiary education degree, and most reported that their spouses were very liberal and open to discussing safe sex practices. The sensitivity of sexual issues could be a possible reason for the poor response rate among lower-educated women, as sexual topics are often shrouded in stigma and social taboos in Asian cultures. Furthermore, participants' responses may also be subject to self-reporting bias due to the sensitive nature of the issues discussed. Therefore, it is essential to interpret the study's findings within the context of its limitations, and researchers should exercise caution when interpreting the results.

This qualitative study provides crucial insights into knowledge gaps and preventive practices, allowing for the development of targeted quantitative research in the future. Consequently, health promotion and education related to ZIKV among pregnant women in Malaysia can be effectively addressed. The positive impact on both qualitative and quantitative studies may potentially influence policymakers in the health sector.

## Conclusions

This study did not identify significant knowledge gaps regarding signs and symptoms of ZIKV infection, and prevention of ZIKV by avoiding mosquito bites among pregnant women in a dengue-endemic country in Southeast Asia who have had tertiary education. However, the study did reveal knowledge gaps in the prevention of sexual transmission of ZIKV, with a small proportion of women demonstrating a lack of knowledge about ZIKV infection during pregnancy and its potential consequences, including congenital abnormalities. Pregnant women in the study population had a low perceived risk of ZIKV infection, likely due to the absence of recent ZIKV outbreaks. Nevertheless, given the rarity of ZIKV infection, it is crucial to educate all pregnant women and their partners about the virus and its prevention. Integrating awareness of STIs during pregnancy into early antenatal care visits is essential to prevent a resurgence of ZIKV infection. The study's findings can inform the development of educational materials tailored to fill knowledge gaps and promote health literacy, ultimately enhancing preventive health behaviors against ZIKV in pregnant women.

### Supplementary Information

**Supplementary Material 1.**

## Data Availability

The datasets used and/or analyzed during the current study are available from the corresponding author upon reasonable request.

## Abbreviations

STI: Sexually transmitted infection
WHO: World Health Organization
ZIKV: Zika virus
