# Supplementary material for: Has Zika been forgotten? A qualitative exploration of knowledge gaps, perceived risk and preventive practices in pregnant women in Malaysia
Source: BMC Womens Health. 2024 Mar 21;24:190. doi: 10.1186/s12905-024-02999-z (PMC10956172; doi:10.1186/s12905-024-02999-z)
Supplement: Supplementary file 1 — Supplementary Material 1. [file 12905_2024_2999_MOESM1_ESM.docx]

**Understanding Zika Virus Infection: Knowledge, Perceived Risk, and Preventive Practices Among Pregnant Women in Malaysia**

In-depth interview guide

| **Theme** | **Questions and probes** |
| --- | --- |
| **Knowledge of Zika** | - Have you come across information about Zika? What details have you encountered, and do you personally know anyone who has experienced Zika? - Can you describe the symptoms of Zika? Are there any additional aspects related to its presentation? - In what manner is Zika contracted? Are there alternative methods of transmission? - If someone were to contract Zika, is there any specific action or treatment that can be taken? - Have you been informed about any potential disabilities linked to Zika? - Are you familiar with the consequences of Zika virus infection during pregnancy, such as the risk of microcephaly? |
| **Worry and perceived severity of Zika** | - What kinds of risks or infections are you concerned about during pregnancy? (e.g., dengue, HIV, hepatitis, COVID-19, etc.) - Do you have concerns regarding Zika? Is there a specific aspect of Zika that you find particularly worrisome? - Are you anxious about the possibility of your baby contracting the Zika virus during pregnancy? - Does the concern about Zika infection extend to worries about the absence of microcephaly development in your baby? |
| **Risk Perception** | - Do you perceive a risk of Zika for yourself? Are you concerned about the potential for Zika affecting your husband or other family members? - Is your home or workplace infested with numerous mosquitoes? - Does your husband work in locations where the risk of mosquito bites is prevalent? |
| **Preventative practices** | - Are you taking any measures to avoid contracting Zika? Could you elaborate on these precautions, including mosquito prevention methods like sleeping under a mosquito net and using insect repellent, as well as strategies for preventing sexual transmission? - Have you engaged in any conversations with your partner regarding the risk of Zika infection? - In the context of discussions about sexual transmission, have you or anyone you're aware of begun using condoms as a preventive measure for Zika? Explore any practices related to sexual and reproductive health for prevention. - How challenging is it to prevent sexual transmission of Zika infection? |
| **Information needs and health promotion** | - Have healthcare workers provided you with any education on Zika during pregnancy? - What specific information would you be interested in learning more about concerning Zika? - What is the most effective method for educating pregnant women about preventing Zika? - Is it more beneficial to educate men about Zika prevention? Why? - Is it considered sensitive to educate pregnant women or discuss sexual transmission and prevention of Zika? |
